# Supplementary material for: Content-rich biological network constructed by mining PubMed abstracts
Source: BMC Bioinformatics. 2004 Oct 8;5:147. doi: 10.1186/1471-2105-5-147 (PMC528731; doi:10.1186/1471-2105-5-147)
Supplement: Additional File 2 — The original results of the above study (non-essential files are deleted to keep the file size under the limit set by BMC bioinformatics). [file 1471-2105-5-147-S2.bz2 › chilibotAdditionalFile2/dip10/659ID10426958E11050/html/BKDA1_BKDA2.html]

 


 **BKDA1** and **BKDA2** 
  
Found 5 abstracts in PubMed, retrieved 5.  
 

 What does Google say? 
 PDF only 
| .edu only 

---

**Interactive relationship** (e.g. stimulation, inhibition, etc)

**Non-interactive relationship** (e.g. studied together, co-existance, homology, etc.)

- Active E1 component of Pseudomonas putida branched chain oxoacid dehydrogenase was purified from P. putida strains carrying pJRS84 which contains bkdR encoding the transcriptional activator and  **bkdA1**  and  **bkdA2**  encoding the alpha and beta subunits.  Ref: 8521848 Eur J Biochem, 1995
- Another construct containing the bkd promoter,  **bkdA1** , and  **bkdA2**  in pKT240 was used to transform P. putida JS113, a mutant which was unable to produce the E1 subunits of the branched chain keto acid dehydrogenase.  Ref: 2211503 J Bacteriol, 1990
- Translation of  **bkdA1**  and  **bkdA2**  from an Escherichia coli expression plasmid produced only 45 kDa and 39 kDa proteins, with N terminal methionine on the 39 kDa protein.  Ref: 8521848 Eur J Biochem, 1995
- Seven genes, previously called yqiS, yqiT, yqiU, yqiV, bfmBAA, bfmBAB, and bfmBB and now referred to as ptb, bcd, buk, lpd,  **bkdA1** ,  **bkdA2** , and bkdB, are located downstream from the bkdR gene in B. subtilis.  Ref: 10094682 J Bacteriol, 1999
- Interestingly, the amount of mRNA specified by the operon ptb bcd buk lpd  **bkdA1**   **bkdA2**  bkdB, which encodes enzymes involved in degradation of branched chain amino acids, also increases after a temperature downshift.  Ref: 12427936 Microbiology, 2002
